# A qualitative study of social connectedness and its relationship to community health programs in rural Chiapas, Mexico

**DOI:** 10.1186/s12889-020-09008-6

**Published:** 2020-06-03

**Authors:** Rachel L. Deitz, Leah H. Hellerstein, Sara M. St. George, Daniel Palazuelos, Trisha E. Schimek

**Affiliations:** 1 Department of Public Health Sciences, University of Miami Miller School of Medicine, Miami, Florida USA; 2 Department of Global health and Social Medicine, Harvard Medical School, Cambridge, MA USA; 3 Contra Costa Regional Medical Center, Martinez, California USA

**Keywords:** Mexico, Social connectedness, Social support, Social isolation, Community health worker, NGO, Community interventions, Qualitative analysis

## Abstract

**Background:**

Social connectedness is an important predictor of health outcomes and plays a large role in the physical and mental health of an individual and a community. The presence of a functioning health clinic with a community health worker program may indirectly improve health outcomes by increasing the social connectedness of the community in addition to providing direct patient care. This study examines the social connectedness of the inhabitants of three Mexican towns within the catchment area of a healthcare Non-Government Organization (NGO) through a qualitative analysis.

**Methods:**

Willing participants were videotaped answering open-ended questions about their community and use of healthcare resources. Interviews were then coded for relevant themes and analyzed for content relating to social connectedness, social isolation, and health.

**Results:**

Respondents reported that having a functioning community clinic had improved their lives significantly through direct provision of care and by reducing the financial burden of travel to seek medical care elsewhere. Respondents from each town differed slightly in their primary means of social support. One town relied more heavily on organized groups (i.e., religious groups) for their support system. Social isolation was reported most frequently by housewives who felt isolated in the home and by respondents that had to deal with personal illness. Respondents that self-identified as Community Health Workers (CHWs) in their respective communities acknowledged that their roles bestowed physical and psychological health benefits upon themselves and their families.

**Conclusions:**

Overall, a long-term health intervention may directly impact the relative social isolation and social connectedness of a community’s inhabitants. The social connectedness of the community is an important quality that must be considered when evaluating and planning health interventions.

## Background

Social connectedness is an important aspect of the human experience and has been shown to play a more significant role than traditional risk factors such as Body Mass Index (BMI) or physical activity in predicting health outcomes [1–4]. Research has demonstrated that interventions focused on reducing social isolation and augmenting connectivity make meaningful differences in health and well-being [5, 6]. Social connectedness, while difficult to define, is present and significant throughout an individual’s lifetime. It relates to the relationships one creates with his or her community and environment, and the support he or she receives in return. It embodies feeling connected to the individuals, community, and environment in which one resides. Social connectedness is closely related to the term social capital, which represents a quantity of connections or resources that one receives through community relationships [7, 8].

While strong ties, or relationships with close friends and family provide social support and increase social capital, a personal and community network rich in weak ties may additionally provide significant contributions to social capital [9]. Weak ties, or loose associations with community members and acquaintances are important for connecting people to different resources within their community [10]. For example, if a community member has met or has association through friends with a town lawyer, doctor, or dentist, they have increased their ability to connect themselves or close family members to health or legal resources to which they may have not otherwise had access. Similarly, the physical infrastructure or built environment of a community also plays a role in its social fabric. If one has access to and familiarity with resources (electricity, roads, schools), he or she can use phones to talk with friends, use roads or sidewalks to reach friends’ homes, or gather with others in parks or other community spaces [11, 12]. Neighborhood social capital may be impacted by common areas such as parks and other green space [13] and can have significant effects on engagement in community health interventions [5]. Other town spaces, such as community-based clinics, provide additional opportunities to augment social capital in a variety of ways [14], conferring both medical and social benefits to those who engage in the clinic program.

Non-government organizations (NGOs) that work in resource-poor areas aim to improve the physical infrastructure of a community through building clinics, wells, or schools. In the case of health interventions, improving health resources often means altering the social context of the community as well, either by better connecting community members to existing providers, or by introducing new providers. Thus, in providing health resources, a health program will impact the social connectedness of the community members it serves. Community health worker (CHW) programs are well set up for this by design, as members of the community are trained to provide health surveillance and screening, and through home visits and community health projects act to connect to and form relationships with other members of the community. CHWs in return increase their own social capital, and find mental health benefits, purpose, and support [15, 16].

The current qualitative study aims to examine the subjective social connectedness of inhabitants in three towns in Chiapas, Mexico, and explore how providing a functioning community clinic with the addition of a CHW program may have impacted the physical and mental health of community members through increasing social connectedness. Specifically, we attempt to understand how the physical clinic, the provision of healthcare, and the interpersonal engagement with clinic staff and CHWs may have conferred social benefits. We also examine how regional characteristics, relationships, and resources may have additional impacts on social connectedness in these communities.

## Methods

### Participants and community characteristics

The communities in the present study, hereafter referred to as Town A, Town B, and Town C, reside in the Sierra Madres de Chiapas of Chiapas, Mexico. While Chiapas has a large indigenous population, the primary language in these three towns is Spanish. Town A is a community of approximately 1200 and is located closest to a larger town with commerce. Its school system provides education through high school. The majority of the inhabitants are of the Jehovah’s Witness faith. Town B is a community of 1800 that is approximately 2 h from the main town. The population is primarily Catholic but with a small community of Protestants. They also offer classes through high school. Town C is the most remote and relatively poorest of the three communities. When road conditions are good, it takes 2.5 h to drive there from the main town. There are approximately 800 inhabitants, most of whom are Catholic. The children in Town C only have the opportunity to study through “telesecundaria,” which offers distance learning through the television for ages 12–15 years. Communication is limited as there is no cell phone reception. Generally, communication with other towns is done through radio or a few telephone landlines that people pay per minute. There is slow wireless internet that can be accessed at the schools, but the network is often overloaded and works the least in the more remote communities. The primary source of income in these communities is coffee farming, with over 90% of the population working in agriculture.

### Existing community health intervention

All three towns have a community clinic run by the same NGO. The clinics were started at various times between 2012 and 2014. These clinics were established and fully functional for at least 1 year prior to the data gathering portion of the study. The NGO ensures that there is one physician staffing the clinic and that it is supplied with medications and equipment. The on-site physician lives in the community and takes care of the inhabitants on a daily basis. These physicians are graduates of Mexican medical schools, often fulfilling their “año de servicio” (year of service). Volunteers and staff from the NGO visit the town and clinic often to perform community outreach, bring supplies and medical equipment, and oversee the clinic function. There are adjunctive programs in place to ensure that patients with complex illnesses have access to specialized studies and care. Each community has a set of female CHWs that act as liaisons between the town doctor and patients with chronic illnesses. The CHW program began in 2013 in Town C and in 2014 in Towns A and B.

### Procedures

In order to learn about and better understand the experiences of community members in an NGO catchment area, interviews were first conducted and videotaped during a three-week period in June of 2015. Videotape was chosen so that interviews could additionally be utilized for a potential internal media project for the NGO to show the impact of the clinic in the communities. Partners Healthcare Institutional Review Board gave an exemption to collect the data as part of a PhotoVoice project, which was considered to be a journalistic endeavor. Participants provided informed consent for use of video footage for both media and research purposes, and had access to the finished project later. The University of Miami Institutional Review Board later granted approval to process these narratives with a qualitative lens and analyze them. Both Institutional Review Boards were provided transcripts of the verbal consent script utilized prior to each interview. Two volunteers affiliated with the NGO canvassed each target community and identified adults (greater than 18 years of age) who were willing to participate. All homes in each of three communities (Towns A, B, and C) were visited. Any adult that was encountered at or around their home during the day and was willing to participate was interviewed. As many men of working age in these towns were out of the house during the day, the data-gathering team more often encountered and interviewed women. Participants were verbally consented and interviewed in their native language of Spanish by a volunteer who was also a native Spanish speaker. Also present during the interview was a second volunteer who was responsible for videotaping the interviews. Participants were told they could end the interview at any time or withdraw their consent for any reason.

Participants were asked five standard questions in an open-answer style format: (1) What is your name and occupation?; (2) What do you do in a normal day?; (3) What is the biggest change you have seen in the town over the past 5 years?; (4) When was the last time you were sick, and what happened?; and (5) What do you do when you feel lonely? Using these open-ended questions, participants were prompted to speak about themselves, their communities, and their use of the clinic. Social connectedness was not specifically mentioned due to challenges related to defining and concisely explaining the term. Furthermore, participants were not specifically asked about social connectedness, social support, or social capital.

Interviews lasted for a minimum of five and maximum of 26 min (mean 11:18 ± 5:01 min). Interviews were kept brief as participants were encountered and interviewed in the middle of their day, causing some interruption to their work, childcare, and other daily responsibilities. If participants offered very brief responses, interviewers included follow-up questions, i.e. “Can you tell me a little more about this?” to encourage further elaboration. Fifty-two interviews were conducted, and two were subsequently excluded due to poor video and/or speech quality. Consent was maintained and not was withdrawn by any of the remaining fifty interviewees for the duration of the research project. Interviews were transcribed, translated from Spanish to English, and coded for themes that relate to social connectedness, including family support, community group support, and social isolation. Overall, 50 interviews were used from the inhabitants of the three towns: 20 from town A, 11 from town B, and 19 from town C.

### Data analysis

We used a general inductive approach [17] for our analysis, by first reviewing the data in its entirety both in video form and through reading transcripts, allowing common themes in the source material to become evident organically. Two coders examined the data in its entirety and developed an initial codebook. We refined the codebook twice prior to the final analysis based on discussions with the research team. To begin the coding process, two coders first individually coded data from one town. Through team discussions, we established consensus on code assignments and defined terms. Once coder agreement was established coders individually coded transcripts from the other two towns. To ensure consistent application of codes, the first author re-reviewed all coded data any time a codebook alteration was made. When there were any questions about the spirit or meaning of the interviewee’s words, we were able to access and re-review the primary source data (video interviews) to gauge a deeper understanding of the interviewee’s intentions. It was determined that data saturation had been achieved given no significant new themes emerged across our 50 interviews based on guidance provided by Guest et al. [18] We performed all transcript coding and data analyses with Dedoose software (version 8.0).

While all data were coded, responses to questions #3 and #5 related to changes in the community and dealing with loneliness were most pertinent to answering the research questions of this study. We drew conclusions based on an overall examination of the social connectedness or perceived social isolation of participants, as well as how structural changes in the community, including the economic situation, physical environment, or access to the local healthcare system may have affected community members’ social connectedness.

## Results

### Demographics

Fifty interviews were analyzed from the inhabitants of Town A (*n* = 20), Town B (*n* = 11), and Town C (*n* = 19). Fifty-eight percent of the interviewees identified as female (*n* = 29) and 42% identified as male (*n* = 21) The two primary jobs identified were “ama de casa” (housewife/homemaker; *n* = 24) and “campesino/agricultor” (farmer; *n* = 16). Two women additionally described themselves as both “ama de casa” (housewife/homemaker) and “acompañante” (CHW). (Table 1).

**Table 1:** Gender, Place of Residence, and Jobs of Study Participants

	*Respondents by Gender*
*Female*	*29*
*Male*	*21*
*Total*	*50*
	*Respondents by Town*
*Town A*	*20*
*Town B*	*11*
*Town C*	*19*
*Total*	*50*
	*Respondents by Job*
*Housewife*	*24*
*Farmer*	*16*
*Retired*	*4*
*Acompañante*	*1*
*Housewife and Acompañante*	*2*
*Student*	*1*
*Mason and Farmer*	*1*
*Nurse*	*1*
*Total*	*50*

### Themes

Three major themes emerged: (1) infrastructure, (2) support, and (3) isolation. Subthemes to infrastructure included mentions of physical changes to the community such as roads, common spaces, and the physical clinic building, as well as the town physician and CHW program, which together provided a healthcare infrastructure for the community. The support theme yielded subthemes where we differentiated between the various types of support utilized and mentioned by community members. Finally, the theme of isolation yielded subthemes which related to the individual’s experience with it; whether their isolation was negatively perceived (i.e., loneliness) or whether it was accepted as a natural part of their life.

### Infrastructure

*In discussions of infrastructure, participants mentioned physical aspects of their communities, including roads, schools, the town square, and the clinic building. The healthcare infrastructure was also widely discussed, including use of the town clinic and mention of the clinic staff, the physician, and the CHW program.*

#### Health clinic

All respondents (n = 50) reported using the clinic at some point, either for themselves or for a family member. When asked generally about community improvements, many respondents (n = 18) identified the town clinic as a positive change. Overall satisfaction with the clinic was evident, with only 2 respondents reporting clinic dissatisfaction. Participants often framed the clinic as a town improvement in the context that it reduced the necessity for extended travel in order to receive treatment, however, it was also mentioned as a physical space for social interaction. One responded remarked when lonely “*...or I’ll go to the clinic, and I’ll see people there to talk to.”* Other expressions included *“We would have to travel far away, but now that the doctors are here, we aren’t traveling, we are not paying so much for medicine. It used to be 1000 pesos every month because we would have to travel all the way to Tuxtla …*” Also, *“Before there weren’t doctors, there wasn’t anything. To see a doctor you would have to go to Jaltenango. Now there is a doctor in the clinic. Now for any pain, you can just go quickly to the clinic. This is the change I’ve seen.”* Community improvements which reduced the necessity for extended travel were significant; in total, 19 interviews were coded with “community improvements to reduce travel,” in which 31 of 33 excerpts referred to local access to medical care. The remaining two excerpts identified a local school and a local market as a community improvement that lessened the burden of lengthy commutes to acquire resources.

#### Community health worker program

The CHW program was frequently described in interviews. As one interviewee described it, *“My spouse is one of the ones that works with them. She receives a monthly compensation of food items for the work that she does. She has achieved control of diabetic patients, of high blood pressure, and other illnesses. This is one of the things in the community that has improved a lot, that has given many people a better life.”* A total of 13 interviewees referred to the CHW program, either when asked about community improvements or in their discussion of healthcare utilization. Respondents from each town mentioned the CHW program: 6 interviewees from town A, 5 interviewees from town B, and 2 interviewees from town C. “CHW Dissatisfaction” was only coded for in one interview with a resident from town C in regards to her first assigned CHW. Otherwise, the response was overwhelmingly positive: *“The second one did visit me, every eight days. She came, we would talk, and I felt happy. I felt content, because of these talks. She wanted to see if I was well.”* Also*, “Yes, Doña []*, *she visits me. We talk about our lives, about how my illness is … when I’m lonely I feel sad because I have no one to talk to. But I talk to the acompañante that visits me or my daughter, and we chat.”*

#### Town resources

Multiple community structures were highlighted as town improvements that made it easier for the townspeople to travel, visit friends and family, and engage more actively in their communities. Respondents in Town A especially made mention of these changes, as their infrastructure had greatly improved over the past 10–15 years. The development of a park, with lights and a walking path, made for an accessible community space that increased interpersonal engagement. *“In the park, lots of people go out and walk now … Before there was nowhere to sit, places for the kids to play, or public lighting … So this wouldn’t allow people to leave their houses to be able to walk, and now the kids have the freedom to play by themselves in the park.”*

In response to question 3, another community member from Town A also mentioned a bridge that had been constructed over a river that separated two small local communities. She explained that the bridge, now a roadway rather than a footbridge, had provided her and her community members with greater opportunities to interact with people from other towns. *“Also they made a bridge in the community, to cross the other side of the river in a car … Now with the bridge, they can pass with their cars to the other side. And they couldn’t do this before … I know more people that come from the other side, and you can see other types of people in the street, from here and from outside.”*

Roadways between the three towns and the nearest city had also improved, making travel easier- impacting access to resources, the local economy, and communication with the outside. Road pavement also improved the ability of townspeople to get around in their individual communities. Interviewees in all three towns highlighted these changes. *“Before the pathways weren’t like now, they were very narrow and difficult … In the past 10 years, now the transportation is better, the life of a farmer is better, we don’t have to transport our products with mules, its much easier to just put it in a car. We don’t have to walk like before, we used to have to walk two hours. This has been a large change, with benefits for everyone, for the whole community.”*

### Social support

*Social support was received by participants through relationships with friends and family members, through a health worker with whom they had formed a particular bond, and through engagement in various community groups. We coded and organized these different types of support accordingly. Frequency of different types of support differed by town, allowing us to extrapolate that inherent differences in each community led the inhabitants to seek support from different areas.*

#### Healthcare worker support

A total of 18 interviewees discussed healthcare worker support, identifying a physician, nurse, or CHW that served an important role in the interviewees life, providing them with a source for social support. Interviewees were not asked to identify persons that they leaned on for support, and were not prompted to discuss their relationships with specific healthcare workers in the community. This topic came up after being asked Question 4, referring to the last time the interviewee was sick, however, the spirit of the sentiments pointed towards a more interpersonal role that the healthcare providers provided: *“But when I talked to the doctor,[ ]… she is the one that encouraged me to go to my appointment. And then with the doctor, that is here now, they attended to me. And they encouraged me to go to my appointments, and they helped me a lot.”* Also: *“The doctors are very nice. Their character is different. I’ve been very content that they have come to work with such enthusiasm, with lots of love.”* After specifically mentioning her doctor by name, one interviewee responded *“I feel good, I have confidence and trust in these doctors, and they get along well with us.”*

#### Community support

Community support (including neighbors and friends) was coded for as frequently as family support (community support n = 20; family support n = 19). Respondents from Town A referred to community members from whom they received support more often than other support systems. Respondents from Town B, in contrast, more often referred to specific healthcare workers that provided social support to them. Community support (in the form of neighbors and friends) was demonstrated via: *“Here in the house, many people visit us. We really like entering into friendships with others, to have friends. My uncle has a saying, that it is more valuable to have 100 friends than 100 pesos in your pocket. So this is like family. And this helps us, to not fall into stress. To always have someone to talk with, to converse with, to pass the time with. Not only in work, but also to dedicate your time with your family and friends.”*

#### Family support

Family support was demonstrated by quotes such as: *“In general I never feel lonely because I have my family with me, I am surrounded by relatives.”* Or “*The truth is, there really isn’t a moment which in I feel lonely. Fortunately, I count on the support of the family, and they are here with me in the community. So I’m not really lonely or alone, I am with them all the time.”* Women described support gained from their children or their in-laws, as they were physically separated from their blood relatives: *“I will go to my mother-in laws house. I don’t have any family here.” “I am not alone because I have my girls, and I dedicate myself to them.”*

### Social isolation

*The theme of social isolation was often introduced as a response to questions 2 and 5, in discussing either a participant’s daily activities or what they did when they felt lonely. Though all townspeople were asked specific questions about dealing with loneliness, some respondents made mention of isolation prior to being prompted to do so. These respondents were either blind or chronically ill (n = 2) or housewives that mentioned feeling isolated in the home (n = 3). In contrast, the majority of male participants did not bring up sentiments of isolation in their interviews. To combat the feeling of loneliness, most interviewees discussed solo activities they would partake in to distract themselves.*

#### Loneliness

Though many interviewees were encountered by themselves in the home, there were a range of sentiments and emotions about their state of aloneness. In coding, we made a distinction between these attitudes. Acceptance was demonstrated by quotes such as *“There’s nothing more to do, than to be here. We don’t feel bad or desperate. No, we are used to it.”* In contrast, some interviewees clearly suffered and felt despair due to being isolated and alone. *“My family is up at the ranch, and I’m here by myself. When the whole family is here it’s happier. But for right now I’m sad and lonely. There’s no one to talk to.”*

Interestingly, to combat loneliness more than half of all respondents (n = 27) discussed that they did activities by themselves to distract from loneliness. These activities ranged from listening music, to watching tv or cooking, to taking a nap. *“I go out. I love music very much. In any moment when I’m alone, I’ll put some music on, or put on a TV program. Or do some personal study of the bible.”*

#### Female isolation in the home

Female isolation was a common subtheme. Ten interviews were coded with “female isolation in the home,” sentiments which referred either to the women themselves being left alone all day (n = 8), or twice mentioned by men in discussing the role of women in general in their community (n = 2). As one (male) interviewee stated, *“You won’t see a woman working because there is nowhere to work. If you do this interview in any other house, you won’t hear 'I have a job.' Here the little girls go to secondary school, but many don’t continue because of the poor economy, or their parents don’t have the opportunity to send them to university, or to have a career, because there is unemployment. So the women stay as housewives. Some maybe learn to sew, or make food, for what I have seen, this is what they reach, and they stagnate.”* Two women noted that unlike their husbands, whose workdays ended at the end of the afternoon, their roles as a housewife did not end and they were expected to either continually clean the home or wait for their husband’s arrival to prepare his meals. When asked how one woman felt in the home, she responded: *“Well sad, but what else am I going to do? I take care of my chickens there, kicking them out of the house so they don’t go to the bathroom on the floor. But what else am I going to do? There’s no one to talk to.”* Similarly*,* another woman noted that her duties at home kept her isolated from her community. *“I don’t go out. Sometimes I see family but I don’t go out because if my husband comes home, who is going to give him his pozol, his drinks? So I don’t go out, I sleep.”* Other women had more accepting attitudes of isolation, stating *“Sometimes I become sad, but now I am accustomed to it.”*

Three of the women who appeared most fulfilled and connected to their communities were the interviewees who identified themselves as CHWs participating in the community health intervention. These women discussed the pride and fulfillment that their roles in the community had brought them: *“So we have seen a great change, because when we began this program, people were very uncontrolled, with very high diabetes, with ulcers, but they are normalizing little by little. And now the majority are controlled. And that makes us, the acompañantes, very happy.”* Another interviewee described her role similarly, noting a sense of connectedness to her community: *“This makes me happy because we are working with this group of acompañantes. For me this is a responsibility, and this makes me happy, because we are supporting others. It makes us take note of other people, and how they are all doing.”*

## Discussion

Through an examination of our qualitative data, we encountered specific examples of how community members felt that the health intervention had altered and improved their communities and themselves over the years. While interviewees made mention of physical health benefits they had gained, many also offered specific ways in which they felt supported by the program and increasingly connected to other community members. The frequency of themes relating to infrastructure and social support made clear that the staffed clinic and CHW program augmented social connectedness through establishment of a stable community structure, provision of equal access to healthcare, and enhancement of interpersonal relationships among healthcare providers, CHWs, and community members.

The town clinic, the cornerstone of the health intervention, was overwhelmingly lauded by the members of each community. Many respondents expressed that a significant attribute of this clinic was that that it reduced the need to travel for both routine and urgent care. This change improved equity among townspeople who no longer needed to rely on their economic status to determine whether they were able to receive healthcare. In addition the individual economic benefit, the reduction in travel meant that time and resources could be spent locally. The presence of the town clinic increased the number of public spaces through which townspeople had contact with other community members, leading to social and mental health benefits [19]. One interviewee even specifically mentioned that when he felt lonely, he would walk over to the clinic to see others. Community engagement was also increased through repeated contact with nurses, doctors, and clinic staff. This may have led to development of additional weak ties between community members and the clinic staff, further facilitating access to additional health resources [20].

The development of more significant connections between community members and clinic staff is well supported through the interviews, as there was often mention of individual staff by name, in contrast to general mention of outside healthcare resources previously relied upon. When respondents discussed previous experiences receiving healthcare, they did not mention specific names, but often listed one, or multiple locations *“we used to have to go to Jaltenango, or Villa Flores, or Tuxtla,”* supporting the idea that respondents had fewer meaningful relationships with healthcare providers or clinics outside of their town to which they would consistently return. In contrast, the participants who mentioned physicians or staff by name demonstrated that they had created meaningful relationships that had provided them with additional social support.

The CHW program provided additional opportunities for meaningful social connection. Interviewees commented on the social aspects of their CHW visits equally or more often than their mention in the context of chronic disease management. Some of the most compelling stories of isolation were told by women that had personal experiences with depression, which they were able to work through by a personal relationship that they formed with a healthcare worker in receipt of treatment. In some cases, the women described their experiences becoming a CHW and helping others as transformative for them. One of the female respondents that discussed being lonely and isolated in the home noted that she was occasionally visited by a CHW for her blood pressure, though she may have benefitted even more by being given the opportunity to become a CHW herself. Benefits to self of becoming a community health worker are noted in the literature by several studies. These benefits include increased knowledge, greater satisfaction, augmented cohesion, increased support and feeling a greater sense of purpose [10, 15, 16, 21].

The means by which the health intervention had an impact on overall social connectedness may in part be explained by and fall within the conceptual framework in gerontology of place attachment [22, 23]. Place attachment, defined by Geographer Rowles, is an individual’s sense of places, made up by the concepts social insideness, physical insideness, and autobiographical insideness. Social insideness refers to the everyday social exchanges in specific places that over time contribute to a person’s sense of community, identity, and belonging. Physical insideness is a person’s subconscious familiarity with the intimate details of a place, such as the shape of cracks in the sidewalk on a walking path. Autobiographical insideness describes how memories and a sense of identity are grounded in a specific place [24]. The NGO intervention created additional places and spaces for community members to use and congregate with others. In addition, by improving the provision of health care with a physical clinic, a town doctor, and community health workers, community members had more time and resources to spend in their local environment. Having this opportunity to participate in daily social exchanges in their local community would allow them to develop and deepen their sense of places and foster their sense of belonging.

Our conclusions about the ways by which the health intervention affected positive social change throughout the target communities is supported by frequent mention of other infrastructural and social group changes, unrelated to the intervention, that had similarly impacted social connectedness. Residents in Town A made mention of the town park, which provided a space for community members to walk and interact. Lighting had been installed and repaired, allowing for safe, common areas for people to walk and congregate together. A bridge had been developed, providing inhabitants of Town A the opportunity to expand their social networks to neighboring inhabitants. Outside of the CHW intervention, interviewees referred to a range of family and social groups that provided support. When stratified by town, differences in individual communities also became clear. The residents of Town A made more mentions of family support, community support, or community group support than in any other town. This may be partially related to the predominance of Jehovah’s Witnesses in this town compared to the other communities surveyed. Many respondents mentioned structured time with their prayer group at least once a week, thus at baseline, this town may have overall been more socially connected, as religious group involvement has been shown to promote civic engagement [25]. Of note, none of the Town C respondents made mention of organized community groups that provided support, providing an opportunity to develop new programs to promote community engagement. These differences highlight the importance of considering the religious observations and level of civic engagement of a town that is targeted for a community health intervention, as CHWs of the same faith and belief system as their patients provide support more effectively [26].

In discussing loneliness, many respondents reported that they would seek out solo activities to distract themselves from how they were feeling, such as reading a book or watching TV. Surprisingly, fewer than half of the interviewees mentioned seeking support from family members, though more mentioned seeking support from the community, for example in mentioning a neighbor or friend. In regards to the female respondents, this may be partially attributed to cultural norms where the wife goes to live with the husband’s family, and thus the housewives had no family close by. The financial insecurity of these women may also have played a role in their social isolation, as is shown by Israel and Farquar et al. [27] and Rankin and Quane [28].

By further examining the social connectedness of these three communities, it may be easier to design and customize interventions to improve the social connectedness of inhabitants within similar communities. For example, residents of Town C, with the lowest identified proportion of support, may benefit from more group interventions, which may strengthen relationships between members of the community. Educational seminars and community outreach may also increase trust in the clinic. Town C additionally is lacking in community green space and divided by a hill, so infrastructural improvements that provide communal areas may confer added benefits. Many residents of Town A mentioned social engagement through their church, so perhaps residents of other faiths within this town may need additional social support. More women may individually benefit from expansion of CHW programs, creating more opportunities for personal involvement, as increases in social networks and new relationships benefit both the patient and CHW.

Though there are a number of benefits to expansion of the CHW program, negative impacts of these changes in the social dynamic of the community must be considered. In the communities we surveyed, women are relied upon to fulfill the role as primary caregiver of their families, and report many responsibilities related to maintaining their homes. Encouraging these women to pursue opportunities to become a CHW may invoke undue stress upon them, decreasing their general health and well-being or creating marital conflict from increased time spent out of the home. Gender differences in the net effects of social capital are well-described. With respect to women, interventions that target social capital may decrease self-rated health [29] or increase depressed mood [30]. The difference in outcomes between men and women may be attributed to societal expectations of gender roles. Moreover, attempts to increase the social connectedness of communities with addiction or intimate partner violence issues may have an unintended effect of normalizing the problems in a community, rather than improving them [31]. Thus, caution must be taken when considering the potential unintended effects of augmenting social capital.

Several aspects of our present findings are significant. Our sample size is relatively large for a qualitative analysis, with substantial one-on one interviews conducted in three different communities in the region. We actively found participants, rather than attempting to elicit response through a survey, and interviews were performed in a setting natural to the community members. Though we coded written transcripts that were subsequently translated into English, the primary data (videotaped interviews) were accessed throughout the coding process, and thus we were able to gain a deeper understanding of the tone and sentiment of the interviewees responses, augmented through use of this type of media. Additionally, we were able to ascertain a great deal about the social connectedness of these communities without incorporating direct questioning on this topic into our study design. By interviewing the residents of these towns via open-ended questions, we obtained insight into the interconnectedness or subjective isolation of individuals and their communities. This study provides insight into the sociological and demographic makeup of this rural region in Southern Mexico, an area rich in cultural and agricultural resources and unique challenges with regards to health and infrastructure. Often studies of long-term health interventions fail to include the intangible aspects of the community, built environment, or social connectedness of its inhabitants as a major mediator or moderator of their success. This qualitative study, in contrast, focuses solely on those aspects and attempts to use the information to inform current and future success of health interventions of these target communities.

There are a number of limitations to our current study that must be considered. As in all qualitative analyses, generalizability is a limitation of the study, as our results are applicable primarily to the particular region, culture, population, and health intervention that we studied. Though the interviews obtained provide ample insight into the shared experiences of the members of these towns, these interviews represent only a small proportion of the inhabitants of each community. The communities in this study are relatively low resource, rural, and geographically isolated, creating unique barriers to a more rigorous data gathering approach that may have yielded a larger sample size. Participants for our study were encountered during daylight hours, which may have reduced the probability that working-age men would have been available and eligible to participate. This is supported by the greater frequency of female interviewees, as they were encountered at home often as the primary caregiver for their young and school-aged children. Additionally, residents of this region are familiar with the only “outsiders” being connected to the healthcare NGO, so they may have assumed that the interviewers were directly related to the clinic project in their area and would have been less likely to provide negative responses out of fear for reduced access or support to their town clinic.

Through these interviews, respondents demonstrated how the addition of a community clinic with CHW program had numerous effects including increased economic stability, community engagement, and personal feelings of safety and support. Respondents also specifically noted how these improvements led to greater social connectedness within their communities, in various ways. They made clear the importance of social connectedness to health and well-being, additionally highlighting other social and environmental changes that had impacted this in their communities. Conversely, respondents who were socially isolated were candid in their negative emotions. This study brings to the forefront the many ways in which long-term interventions impact the communities in which they aim to serve, and highlights the social and societal impacts that may not be initial aims of the interventions.

## Conclusion

Though the benefits of social connectedness are well-known [15, 28–31], long-term health interventions often focus on measurable benchmarks (systolic blood pressure, hemoglobin A1c) and either neglect to consider the role of interconnectedness, or to simply refer to it in the literature as a potential mediator. Beyond a mediating factor, health interventions often incidentally increase the social connectedness of the patients they serve- linking patients to community resources, improving communication between community members, or serving as a direct caregiver. When CHWs are utilized, they not only provide support and increase the sense of well-being in their patients, they often form meaningful relationships with them [21, 26, 32–36]. Research that evaluates the social connectedness of individual communities prior to and following health interventions is necessary to determine how these programs can augment the social connectedness of their target communities, thereby improving the health of their inhabitants. Looking ahead, we recommend that for advance planning of future health interventions in rural communities, NGOs and other health organizations assess and apply strategies to improve the social connectedness of the communities they plan to serve, and consider these needs in addition to the physical health needs of their target communities. By recognizing how social connectedness impacts health, long-term health interventions can have even greater impacts on the individuals and communities they serve.

## Data Availability

The datasets used and/or analyzed during the current study are available from the corresponding author on reasonable request.

## Abbreviations

NGOs: Non-government organizations
CHW: Community health worker
